# Psychedelics and mental health: reimagining care through science, insight, and compassion

**DOI:** 10.3389/fphar.2025.1649929

**Published:** 2025-09-03

**Authors:** Georgios Mikellides, Marios Kyriazis

**Affiliations:** ^1^ Cyprus rTMS, Larnaca, Cyprus; ^2^ Department of Basic and Clinical Sciences, Medical School, University of Nicosia, Nicosia, Cyprus; ^3^ National Gerontology Centre, larnaca, Cyprus

**Keywords:** psychedelics, ketamine, MDMA, psilocybin, LSD, rTMS

## Introduction

In recent years, psychiatry has witnessed a renaissance in the investigation of psychedelic compounds, a broad class of psychoactive substances that induce altered states of consciousness, often characterized by changes in perception, mood, and cognition, which were largely shelved following regulatory crackdowns in the 1970s. The renewed interest is driven by a mounting crisis in mental health treatment efficacy, particularly for conditions labelled as treatment-resistant. As research continues to underscore the limitations of conventional monoaminergic antidepressants, there is a growing openness to alternative paradigms that were previously considered fringe (Wang and Wang, 2025).

These substances can be categorized into Classic psychedelics (Serotonergic hallucinogens, such as LSD -Lysergic acid diethylamide, psilocybin, and mescaline), Entactogens, such as MDMA (3,4-methylenedioxymethamphetamine), and Dissociative psychedelics such as ketamine and phencyclidine (PCP).

Psychedelics are being revisited not merely as pharmacological agents but as facilitators of profound psychological and existential experiences with therapeutic implications. This opinion article explores the current state of evidence, discusses the mechanisms by which psychedelics may exert their effects, and advocates for a balanced, evidence-based integration of these compounds into modern psychiatric care.

### Therapeutic potential and current evidence

Among the most compelling findings is the rapid and enduring antidepressant effect of psychedelics, often after just one or two sessions, especially when combined with psychotherapy, but it is also unclear how much of the effect has to do with the therapy. Increasingly, studies are being conducted with minimal or no therapy (Li and Wang, 2025). Psilocybin has demonstrated significant efficacy in reducing depressive symptoms in several randomized controlled trials (RCTs). Carhart-Harris et al. (2021) reported that in this 6-week randomized study comparing psilocybin and escitalopram for individuals with persistent mild-to-severe depression, there was no significant difference between the two groups in the primary outcome—the change in QIDS-SR-16 depression scores at week 6. Although secondary outcomes tended to favour psilocybin, the results should be interpreted with caution, as the confidence intervals were not corrected for multiple comparisons, making firm conclusions unreliable. Both groups showed lower depression scores at week six compared to baseline, but the lack of a placebo group makes it difficult to determine how much of the improvement was due to the treatment itself. Here, and as in the case of MDMA trials below, we acknowledge the need for an ‘active placebo’ use with low-dose psychedelics, in order to avoid unsuccessful blinding (Butler et al., 2022; Wen et al., 2024).

Similarly, MDMA-assisted psychotherapy has shown impressive results in the treatment of PTSD (Post-Traumatic Stress Disorder). Unlike classic psychedelics such as psilocybin or LSD, which act primarily as 5-HT2A (5-hydroxytryptamine 2A) receptor agonists, MDMA operates through a different mechanism. It increases synaptic levels of serotonin, norepinephrine, and dopamine, and promotes the release of oxytocin, which may underpin its empathogenic and prosocial effects. The Phase 3 trial by Mitchell et al. (2021) demonstrated that MDMA significantly reduced PTSD symptoms and functional impairment compared to placebo, with a large effect size and favourable safety profile and yet, this was declined by the FDA in the summer of 2024. These critiques suggest that psychedelic therapy must meet not only efficacy thresholds but also stringent expectations for rigor, transparency, and real-world feasibility. This tension highlights the evolving regulatory landscape and the need for further multisite trials that balance scientific enthusiasm with methodological conservatism.

### Mechanisms of action: beyond monoamines

Psychedelics primarily exert their effects through agonism at the serotonin 5-HT2A receptor, leading to a cascade of neurobiological changes. These include increased synaptogenesis, neuroplasticity, and altered connectivity between key brain networks such as the default mode network (DMN), salience network, and executive control network (Vollenweider and Preller, 2020). The transient disintegration of the DMN, often associated with the ego-dissolution experience, appears to facilitate a cognitive reset, allowing patients to revisit entrenched thought patterns with a renewed perspective. This mirrors findings in ketamine research, where rapid antidepressant effects are also linked to neuroplasticity and the reconsolidation of emotional memory (Mikellides et al., 2022). Moreover, psychedelics enable emotional catharsis, insight, and existential re-evaluation, phenomena that are often absent in pharmacotherapy based on chemical symptom suppression. Mikellides et al. (2024) describe the experience as a form of psychological catharsis—a profound emotional release. While some practitioners draw parallels between catharsis and non-ordinary states of consciousness, it is important to distinguish this from a true spiritual emergency. As defined by Grof & Grof, spiritual emergency is more akin to a transient psychotic-like experience marked by identity disruption and disorientation, not merely emotional release (Grof and Grof, 1989; Lukoff et al., 1998). Conflating the two may inadvertently suggest that destabilization is necessary for healing, a notion that carries clinical and ethical risks.

### Clinical integration and challenges

Preliminary evidence suggests that the therapeutic context—including set, setting, and therapist support—may influence outcomes with psychedelics. The ‘set’ refers to the patient’s mindset, expectations, intentions, and mental state going into the session; the ‘setting’ involves the physical and social environment in which the psychedelic is administered. (Gorman et al., 2021; Ko and Lewis, 2022). However, the specific contribution of these factors remains unclear in the absence of controlled studies directly comparing drug administration with and without such support.

These substances are not stand-alone medications; rather, they function as adjuncts to intensive psychotherapeutic processes. Training therapists and establishing ethical, evidence-based protocols is essential. Additionally, psychedelic treatments remain inaccessible for many due to cost, regulatory hurdles, and stigma and a big portion of the cost is likely to be the labor required to do the therapy (Marseille et al., 2022). These authors demonstrated that MDMA-assisted therapy is not only clinically beneficial but also cost-effective for PTSD treatment. Despite high up-front costs, the intervention yielded substantial long-term savings and health improvements, especially when delivered with a third session.

These findings suggest that MDMA-AT offers substantial clinical benefits and is cost-saving from a payer’s perspective for patients with severe PTSD. However, the authors acknowledge that further research is needed to confirm these results and address potential limitations. Current clinical trials are often conducted in highly controlled academic environments, which may limit generalizability. However, naturalistic studies, such as those involving ketamine (Mikellides et al., 2022), provide encouraging evidence of real-world effectiveness in community settings.

### Comparison with ketamine and other non-conventional treatments

Ketamine’s entry into psychiatric practice has paved the way for psychedelics. As a dissociative anaesthetic with glutamatergic activity, ketamine produces rapid antidepressant effects in patients with Treatment-Resistant Depression (TRD). It has established a precedent for rethinking what constitutes an effective intervention and challenged the dominance of the monoamine hypothesis (Mikellides et al., 2023). The comparison of ketamine with repetitive transcranial magnetic stimulation (rTMS) in TRD, as explored by Mikellides et al. (2022), who highlight that non-traditional therapies, such as ketamine-assisted psychotherapy, demonstrate both safety and efficacy in treatment-resistant populations. Ketamine induces altered states of consciousness that have shown transformative psychological effects in some patients. Building on this, the field is now exploring classic psychedelics like psilocybin and LSD, which may offer even deeper transpersonal experiences and long-lasting therapeutic benefits.

### Combination therapy: psychedelics and rTMS

Emerging anecdotal and clinical evidence (Dębowska et al., 2023) suggests that combining psychedelics with neuromodulatory interventions like rTMS may yield synergistic effects. rTMS, a non-invasive brain stimulation method targeting specific cortical regions, is known to modulate neural circuitry and improve symptoms of depression, OCD (Obsessive-Compulsive Disorder), and PTSD. In clinical settings in Cyprus rTMS Centres has been used for over 6 years, with growing interest in integrating it with psychedelic-assisted psychotherapy. The hypothesis is that rTMS may enhance neuroplasticity. This integrative model represents a frontier in personalized psychiatry and warrants formal study. The combined use of ketamine and rTMS (with the Mikellides Protocol) has promising clinical potential, as demonstrated by us (Saabneh et al., 2025; in Press).

### Psychedelics and existential distress in end-of-life care

Beyond depression and PTSD, psychedelics have shown promise in alleviating existential distress in terminally ill patients. Grob et al. (2011) and Griffiths et al. (2016) found that psilocybin significantly reduced anxiety and depression in patients with life-threatening cancer diagnoses. Participants described enhanced meaning, acceptance of mortality, and spiritual wellbeing following psychedelic-assisted therapy.

### Comparative safety and misconceptions

Public and professional scepticism about psychedelics often centres on safety concerns. However, accumulating evidence suggests that, within controlled environments, psychedelics in review are physiologically safe and non-addictive (Zhang et al., 2025, Behera et al., 2024).

Gasser et al. (2022) found that classic psychedelics have an exceptionally low toxicity profile and negligible risk of dependence. Adverse events are rare and usually relate to unsupervised use or underlying psychotic vulnerability. Proper screening, preparation, and integration mitigate these risks. In fact, some experts argue that the relative safety of psychedelics compares favourably with commonly prescribed psychiatric medications.

### Neuroethical reflections and the question of identity

One of the most intellectually provocative aspects of psychedelic therapy is its impact on identity, belief systems, and metaphysical assumptions. Reports of ego dissolution, mystical experiences, and spiritual insight raise questions about neuroethics and informed consent (Letheby, 2021). While many patients find these experiences beneficial, some may find them disorienting or even distressing (Yaden and Griffiths, 2021). Historical and even recent abuses, underscore the potential for misuse of psychedelics.

### The role of psychotherapy in psychedelic-assisted treatment

The integration of psychotherapy with psychedelic administration has been central to most clinical trials to date. Although many protocols include structured therapeutic support before, during, and after psychedelic sessions, there is a lack of rigorous randomized trials directly comparing drug-alone *versus* drug-plus-therapy conditions. This limitation makes it difficult to determine whether psychotherapy is essential or simply supportive. A recent commentary underscores this uncertainty. Zamaria et al. (2025) argue that while therapy is widely used alongside psychedelics, its specific therapeutic contribution has not been empirically delineated. Goodwin et al. (2023) similarly question whether the benefits observed in psilocybin trials are due to the drug itself or to the psychotherapeutic framework in which it is delivered. Naturalistic studies of ketamine treatment provide useful insights. For example, Dore et al. (2019) reported significant clinical improvements in patients receiving ketamine-assisted psychotherapy, yet noted that similar outcomes have been observed in patients receiving ketamine infusions without formal therapy. Additionally, Joneborg et al. (2022) highlighted transient neural changes associated with ketamine treatments, suggesting that pharmacological effects alone may drive significant portions of the therapeutic response.

### Toward a new therapeutic paradigm

The resurgence of psychedelic psychiatry suggests a turning point in the field. These substances urge us to reconsider the role of consciousness, meaning, and personal narrative in healing. In contrast to the reductionist view of mental illness as mere chemical imbalance, psychedelics promote a reintegrative model where subjective experience, relationship, and insight are central. Such a shift aligns with broader calls in the psychiatric community to rehumanize care. It echoes the principles of compassionate, trauma-informed, and relational psychiatry, where healing arises not solely from neurochemical modulation but through connection, presence, and personal transformation (Mikellides et al., 2023).

### The path forward: recommendations

To responsibly harness the benefits of psychedelics, we recommend the following.

1. Expand Research: Larger, multisite, placebo-controlled trials are needed to refine protocols and determine long-term efficacy and safety. However, it needs to be clarified that in psychedelic research it may be best to use low-dose psychedelics as an active placebo.
2. Therapist Training: Establish more widely accessible accredited training pathways for psychedelic-assisted therapists such as MAPS (The Multidisciplinary Association for Psychedelic Studies) emphasizing ethics, trauma sensitivity, and integration skills.
3. Policy Reform: Advocate for policies that support compassionate access while upholding rigorous medical standards.
4. Public Education: Combat stigma through evidence-based dialogue that frames psychedelics as tools of healing, not recreation.
5. International Collaboration: Learn from global traditions and practices

## Conclusion

Psychedelics are re-entering psychiatry not as recreational relics of the 1960s but as scientifically credible, clinically relevant treatments for some of the most challenging conditions in mental health. Their potential to accelerate healing, enhance insight, and restore meaning makes them uniquely suited to address the deep suffering that characterizes treatment-resistant disorders. As with any powerful intervention, psychedelics demand caution, respect, and a framework of ethical use. But if integrated wisely, they may help usher in a new era of psychiatry—one that prioritizes not only symptom remission but personal transformation, relational depth, and spiritual wellbeing. The path forward is not merely about approving new molecules. It is about redefining what it means to heal.
